# Simulation Analysis and Comparison of New Hybrid TLI-µTESLA and Variant TESLA Protocols Using SHA-2 and SHA-3 Hash Functions

**DOI:** 10.3390/s22239063

**Published:** 2022-11-22

**Authors:** Khouloud Eledlebi, Ahmed Adel Alzubaidi, Chan Yeob Yeun, Ernesto Damiani, Victor Mateu, Yousof Al-Hammadi

**Affiliations:** 1 Center for Cyber-Physical Systems, Khalifa University, Abu Dhabi P.O. Box 127788, United Arab Emirates; 2 Department of Electrical Engineering and Computer Science, Khalifa University, Abu Dhabi P.O. Box 127788, United Arab Emirates; 3 Technology Innovation Institute, Abu Dhabi P.O. Box 9639, United Arab Emirates; 4 Cryptography and Graphs Group, Universitat de Lleida, 25001 Lleida, Spain

**Keywords:** continuous authentication, scalability, cybersecurity, hash function, immediate authentication, low overhead, TESLA protocol

## Abstract

The evolution of 5G and 6G networks has enhanced the ability of massive IoT devices to provide real-time monitoring and interaction with the surrounding environment. Despite recent advances, the necessary security services, such as immediate and continuous authentication, high scalability, and cybersecurity handling of IoT cannot be achieved in a single broadcast authentication protocol. This paper presents a new hybrid protocol called Hybrid Two-level µ-timed-efficient stream loss-tolerant authentication (Hybrid TLI-µTESLA) protocol, which maximizes the benefits of the previous TESLA protocol variants, including scalability support and immediate authentication of Multilevel-µTESLA protocol and continuous authentication with minimal computation overhead of enhanced Inf-TESLA protocol. The inclusion of three different keychains and checking criteria of the packets in the Hybrid TLI-µTESLA protocol enabled resistance against Masquerading, Modification, Man-in-the-Middle, Brute-force, and DoS attacks. A solution for the authentication problem in the first and last packets of the high-level and low-level keychains of the Multilevel-µTESLA protocol was also proposed. The simulation analysis was performed using Java, where we compared the Hybrid TLI-µTESLA protocol with other variants for time complexity and computation overhead at the sender and receiver sides. We also conducted a comparative analysis between two hash functions, SHA-2 and SHA-3, and assessed the feasibility of the proposed protocol in the forthcoming 6G technology. The results demonstrated the superiority of the proposed protocol over other variants in terms of immediate and continuous authentication, scalability, cybersecurity, lifetime, network performance, and compatibility with 5G and 6G IoT generations.

## 1. Introduction

The advances in 5G technology and the expansive research in 6G technology have led to a potential increase in the scalability of IoT networks by a factor of 500 compared to the existing 4G technology. The recent progress in this direction helps achieve transmission rates that are 10 times greater than those in the LTE network [1,2]. The expansion of the IoT network is the primary reason why constrained devices, such as sensors, RFIDs, and other smart devices, are likely to play a significant role in accessing and transferring essential information [3,4,5]. This, however, increases the vulnerability of the IoT networks, leading to a higher probability of malicious attacks, both external attacks as well as those within the network. This is mainly because of the limited energy sources and communication bandwidth of constrained devices, which further restrict the type of security protocols that can be used to protect the network [6].

The lightweight cryptography protocols are simplified encryption protocols that involve relatively low computational complexity, and they are often used in constrained IoT devices to enable sufficient security services considering their limited resources [7,8,9,10]. An important lightweight broadcast authentication protocol called the timed-efficient stream loss-tolerant authentication (TESLA) protocol allows confidentiality, integrity, and user authentication because it integrates sufficient cryptographic functions and processes while maintaining low communication and computation overhead in constrained devices [11,12]. TESLA protocol and its variants rely on symmetric cryptography, including symmetric keys that are used to authorize the broadcasted packets, and the generation of the MAC value to authenticate the integrity of the packets [13]. The TESLA protocols also rely on asymmetric cryptography mainly because of the loose synchronization between the sender and receiver as well as the introduction of a disclosure time delay that is aimed at protecting the symmetric keys. The main limitations associated with the TESLA protocol are as follows: inability to support the scalability of IoT devices, authentication between the sender and receiver, immediate authentication of the user and its packets, and vulnerability to DoS and Brute-force attacks. The previous limitations are critical targets that should be maintained in the IoT network within the 5G and 6G technologies to enable real-time monitoring and transfer of data among the members and to ensure real-time communication with the surrounding environment by keeping the network updated.

To address the limitations associated with the original TESLA protocol, several improvement-based approaches were proposed. However, no single approach could help overcome all the limitations at once. A primary example of such an approach is the Infinite-TESLA (Inf-TESLA) protocol, which was introduced to reduce the computation overhead in the network and increase the security of the keychains by introducing two parallel keychains to authenticate the transmitted packets [14]. It was suggested that the Inf-TESLA protocol could also provide continuous authentication between the sender and receiver by relying on a single synchronization that occurs while establishing the communication bandwidth between the parties. However, this was proven wrong in [15], wherein it was stated that the continuous authentication is constrained by the length of the two keychains, and a new synchronization between the sender and receiver is required. Hence, the enhanced Inf-TESLA protocol was introduced to provide a continuous authentication between the sender and receiver during the time they are connected, and we only relied on symmetric encryption and one-way hashing processes, which do not increase the computation overhead of the network. However, neither the Inf-TESLA nor the enhanced Inf-TESLA protocols support scalability nor immediate authentication of IoT devices because their structure is somewhat similar to the original TESLA protocol.

The µTESLA protocol was introduced to simplify the authentication process and reduce the buffer overflow in the network by unicasting the broadcast authentication to each receiver at a time [16]. The protocol reduced the scalability of the network because it restricted the total number of receivers connected to the system. Hence, the Multilevel-µTESLA protocol was introduced to address the scalability problem in the µTESLA protocol by introducing two-level keychains: a high-level keychain that is directly related to the sender and covers the lifetime of the receiver and low-level keychains associated with each time interval of the high-level keychain [17]. The short time intervals in the low-level keychains nearly achieved immediate authentication between the sender and receiver and reduced the DoS and Brute-force attacks. However, the Multilevel-µTESLA protocol does not support continuous authentication between the sender and receiver, as it requires a new synchronization at the end of each low-level keychain.

The previous limitations in the original and variant TESLA protocols motivated us to develop a hybrid protocol called Hybrid TLI-µTESLA (Hybrid Two-level µTESLA) protocol that can combine and maximize the benefits of the previous protocols, while maintaining an acceptable level of computation and communication demands in the network. Hence, the primary motivation of this study is that the Hybrid TLI-µTESLA protocol combines the Multilevel-µTESLA structure, possessing a high-level and low-level keychain, along with the enhanced Inf-TESLA protocol by including two parallel low-level keychains that operate in an alternating mode. The high-level keychain possesses relatively long time intervals to cover the lifetime of the receivers and reduces the computation overhead of the network. Additionally, the two low-level keychains, ensures a continuous authentication between the sender and receiver, without requiring unnecessary synchronization that may potentially drain the energy and reduce the lifetime of the network. The low-level keychains involve considerably short time intervals to achieve immediate authentication between the parties and reduce the Brute-force and DoS attacks. In other words, the Hybrid TLI-µTESLA protocol could seamlessly realize the scalability of IoT devices with nearly immediate and continuous authentication, and enhance the cybersecurity of the network through the inclusion of three different keychains while maintaining an acceptable level of computation and communication demands.

We also highlighted the authentication problems of the first and last high-level and low-level packets in the Multilevel-µTESLA protocol and proposed an efficient solution. Finally, we analyzed the impact of the change in the hash function from SHA-2 to SHA-3 on the overall performance and authentication process of the Hybrid TLI-µTESLA and other variant TESLA protocols. Based on these factors, we verified the feasibility of the proposed Hybrid TLI-µTESLA protocol in handling quantum attacks and operating efficiently within the acceptable level of computational requirements for the upcoming 6G technology. The contributions of this study are summarized as follows:

1. Developing Hybrid TLI-µTESLA protocol that combines the benefits of all previous variant TESLA protocols, including scalability, immediate and continuous authentication, and high security level while maintaining an acceptable level of computational and communication demands.
2. Addressing the authentication problem of the first and last packets of the high-level and low-level keychains in the Multilevel-µTESLA protocol and subsequently proposing a minimal computational overhead solution.
3. Analyzing the time complexity of the Hybrid TLI-µTESLA and other variant TESLA protocols through simulation results and curve fitting.
4. Performing comparative analysis of the Hybrid TLI-µTESLA and other variant TESLA protocols in terms of the computational overhead on the sender and receiver sides.
5. Considering comparative analysis of the Hybrid TLI-µTESLA and other variant TESLA protocols in terms of changing the hash function from SHA-2 to SHA-3 and monitoring the overall computational overhead on the sender and receiver sides.
6. Studying the robustness of the Hybrid TLI-µTESLA protocol against cybersecurity attacks including masquerading, modification, man-in-the-middle, Brute-force, and DoS attacks.

The remainder of this paper is organized as follows. Section 2 discusses a review of a variant TESLA protocol wherein a comparison of our Hybrid TLI-µTESLA protocol with other variant protocols is done. Section 3 explains the construction of the Hybrid TLI-µTESLA protocol in detail. Section 4 highlights the unaddressed problem of authenticating the first and last packets in the low-level and high-level chains in the Multilevel-µTESLA protocol and the proposed solution that is later implemented in the Hybrid TLI-µTESLA protocol. Section 5 provides a comparative analysis of the Hybrid TLI-µTESLA and other variant TESLA protocols. Finally, Section 6 summarizes the conclusions and implications of the study.

## 2. Related Work

This section discusses the latest variants of TESLA protocols that are used to compare our proposed Hybrid TLI-µTESLA protocol with. A detailed explanation about all the variants is given in [18].

### 2.1. Original TESLA Protocol

TESLA is a lightweight broadcast authentication protocol that can be applied to constrained devices to protect the transferred packets between the sender and receiver while maintaining an acceptable level of computation and communication overhead [11,19]. The functionality of TESLA relies upon unique properties that require the usage of combined symmetric and asymmetric cryptographic functions. The symmetric property is achieved by using symmetric keys within a keychain generated using one-way hash function with the aim of protecting the transmitted messages at the sender end and generating the MAC values using the messages and the keys to be sent to the receiver [7,9]. On the other side, the receiver should have the same key that is used by the sender to generate the MAC values, and compare it with the value embedded in the message to guarantee the confidentiality, integrity, and authentication of sender and the message. The asymmetric property in TESLA protocol is achieved by using asymmetric cryptography/digital signatures to bootstrap new receivers and share the symmetric keys between the two parties. Moreover, a delay time interval (d) is introduced to keep the symmetric key hidden from the receiver before it is retransmitted for authentication purposes. The TESLA protocol also relies on loose time synchronization between the sender and receivers to reduce the complexity of the hardware design and reduce any delay in the communication between devices.

The limitations associated with the TESLA protocol are related to the lack of scalability of new IoT devices joining the network and high probability of losing the predefined keychain packets due to weak communication. It further results in wastage of time and energy in establishing synchronization with the same sender owing to the expiration of the keychain during the communication time window associated with the receiver. Moreover, immediate and continuous authentication are not supported by TESLA protocol. Therefore, several upgrades to the original TESLA protocol were proposed to improve the cybersecurity, connectivity, and scalability.

### 2.2. INF-TESLA and Enhanced INF-TESLA

In the original TESLA protocol, when the keychain reaches the last key element, the system needs to reestablish synchronization between the same sender and receiver as if they are new to the connection. Such unnecessary establishments may increase the computational demands and power consumption. To solve this problem, the Inf-TESLA protocol introduced two parallel key chains that are in offset alignment with each other. This alignment ensured that if one keychain has come to an end, the other keychain still keeps the synchronization between the sender and the receiver working [14]. The functionality of the two keychains can follow either a two-key mode, where both keys are sent in the packet, or an alternating mode, wherein a key from either chain is presented alternatively in the packet, such that one keychain is responsible for the odd time intervals and the other is responsible for the even time intervals.

The Inf-TESLA protocol claimed that continuous synchronization between the sender and receiver is guaranteed for the entire lifetime of the network. However, in [15], it was shown that the Inf-TESLA protocol continuity is limited to the length of the first two keychains. In other words, once the two keychains expire, the sender should reestablish synchronization with the same receiver, which results in a significant wastage of energy, memory space, and time. Hence, the enhanced Inf-TESLA protocol was developed to improve the Inf-TESLA protocol, which allows continuous communication and authentication between the sender and receiver by reconstructing new offset keychains during their communication time window without the need for terminating the connection and determining a new synchronization [15]. The continuous authentication in the enhanced Inf-TESLA protocol depends on the symmetric encryption of the new exchanged commitment keys between the sender and receiver at the last time intervals of the existing keychains. The symmetric encryption is performed using a master key that is exchanged between the sender and receiver in their first synchronization packet. A detailed explanation about the process of the enhanced Inf-TESLA protocol is given in [15]. The limitations of the Inf-TESLA and enhanced Inf-TESLA protocols include lack of immediate authentication and scalability of IoT devices, mainly because their structure is similar to that of the original TESLA protocol.

### 2.3. µTESLA and Multilevel-µTESLA Protocols

The µTESLA protocol is aimed at simplifying the functionality of the original TESLA protocol by unicasting the packets to each receiver individually [16]. Rather than adding a disclosed key to each data packet, the key disclosure is sent once per time interval, and it is independent of the broadcasted packets. This process reduces the receiver’s computational power and occupation of the communication bandwidth with unnecessary packets. Because the number of legitimate receivers is limited in the µTESLA protocol, one-way keychain will not be stored in the receiver memory, which limits the scalability of the system. To overcome this, several approaches to improve the µTESLA protocol were proposed, including the Multilevel-µTESLA protocol. The main advantages of this protocol are the reduction in the authentication time delay between the sender and receiver, and the reduction in the probability of denial of service (DoS) attacks [17].

**Figure 1:**
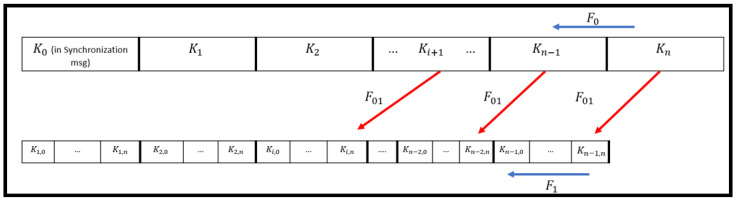
General Structure of Multilevel-µTESLA Protocol.

The Multilevel-µTESLA protocol generates two keychain levels, a high-level keychain, that is directly connected to the sender, and low-level keychain, that is responsible for authenticating the transferred packets between the sender and receiver. The high-level keychain has long time intervals to cover the lifetime of the receiver without requiring establishment of a new keychain, which reduces the computational complexity of the network. Each time interval in the high-level keychain is further divided into significantly short time intervals that correspond to low-level keychains. The short time intervals reduce the time required to receive and authenticate the packets, which further reduces the probability of having a DOS attack. Figure 1 illustrates the deployment of the two keychain levels of Multilevel-μTESLA protocol. A notable factor associated with Multilevel-µTESLA protocol is that the low-level keychain is connected to the high-level keychain such that the low-level keys can be generated from the high-level keys using one-way hash function in instances where some low-level packets are lost. In other words, the last element in the low-level keychain is the result of the implementation of a pseudo-random function on the high-level key related to one time interval before the interval for which low-level keychain is generated, as shown in Figure 1. The generation of the low-level key from the high-level key is represented in Equation (1) as follows:

(1) $$K_{i,n}=F_{01}\left(K_{i+1}\right)$$

where $K_{i,n}$ is the last nth element of the low-level keychain in the ith time interval, $F_{01}$ is the pseudo-random function, and $K_{i+1}$ is the high-level key in the (i+1) time interval. The Multilevel-μTESLA protocol introduces an additional authentication message called commitment distribution message (CDM), which is used to send the commitment keys of the low-level keychains that should be generated in the time intervals of the high-level keychain. The construction of the CDM packet is represented in Equation (2).

(2) $$CDM_{i}=i\left|\;K_{i+2,0}\;\right|MAC_{K_{i}}\;(i\;|K_{i+2,0})\;|\;K_{i-1}\;$$

where $CDM_{i}$ is the CDM packet sent during ith time interval, $K_{i+2,0}$ is the commitment key of the low-level keychain used in (i+2) time interval, $K_{i}$ is the high-level key of ith time interval, $MAC_{K_{i}}$ is the MAC value generated using $K_{i}$ key to protect the commitment key $K_{i+2,0}$, $K_{i-1}$ is the high-level key of the (i−1) time interval used to authenticate the previously buffered $CDM_{i-1}$ packet at the receiver side, and | is the concatenation process.

It should be noted that the CDM packet usually sends the commitment key of the low-level keychain related to two time intervals ahead to ensure that the receiver has sufficient time to buffer and authenticate the important information before receiving the corresponding packets [17]. To avoid loss of important information that is sent by the CDM packets, the sender should randomly send several copies of the $CDM_{i}$ packet during the ith interval without having a regular timing pattern between the copies. The drawback of the Multilevel-µTESLA protocol is that it does not support continuous authentication within the network members.

The previous discussions showed that a specific TESLA protocol variant is capable of combining the immediate and continuous authentication with increased cybersecurity level, within acceptable level of computational demands. Hence, the structure of the proposed Hybrid TLI-µTESLA protocol is a combination of Multilevel-µTESLA protocol, with the inclusion of high-level and low-level keychains responsible for immediate authentication, and the enhanced Inf-TESLA protocol, with two parallel keychains implemented in the low-level keychain responsible for continuous authentication and reduction in the computational demands. The new structure of the Hybrid TLI-µTESLA protocol helps in achieving high level of cybersecurity, while maintaining low computational demands at the sender and receiver sides. The next section explains the construction of the Hybrid TLI-µTESLA protocol in detail.

## 3. Proposed Hybrid TLI-µTESLA Protocol

The TLI-µTESLA protocol was introduced theoretically in [20] by merging the Multilevel-µTESLA protocol and Inf-TESLA protocol to achieve lower computation and continuous authentication. However, in [15], it was proven that the claimed continuous authentication in the Inf-TESLA protocol is limited to the duration of its two keychains. Consequently, we developed a novel variant of Inf-TESLA, referred to as the enhanced Inf-TESLA protocol, in [15]. This protocol allows a seamless connection and authentication of the transmitted packets between the sender and receiver by regenerating new offset keychains during their communication time window, without the need to re-establish synchronization packets. In this study, we propose an approach to modify the TLI-µTESLA protocol by merging the Multilevel-µTESLA protocol with the enhanced Inf-TESLA protocol, and we refer to it as the Hybrid TLI-µTESLA protocol.

**Figure 2:**
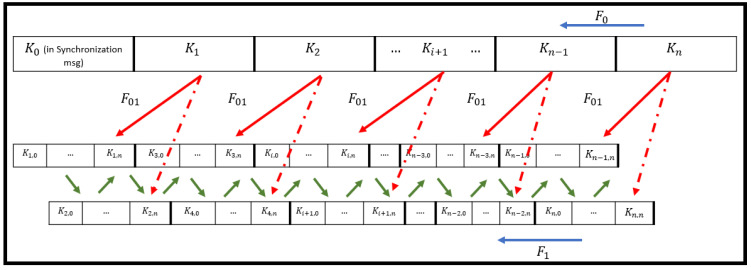
General Structure of the proposed Hybrid TLI-µTESLA Protocol.

The new contribution in the Hybrid TLI-µTESLA protocol is the addition of two continuous low-level keychains instead of one low-level keychain in the Multilevel-µTESLA. This ensures a continuous synchronization between the sender and receiver during the receiver lifetime. Figure 2 shows the structure of the Hybrid TLI-µTESLA protocol low-level and high-level keychains, wherein the green arrows represent the alternating mode used in the two low-level keychains following the enhanced-Inf TESLA protocol. The generation of the Hybrid TLI-µTESLA protocol is initiated with the construction of the High-level keychain at the sender side, wherein the last key element of the chain, *K_n_*, is randomly chosen and the hash function *F*_0_ is applied for L number of times; where L is the length of the high-level keychain. The time window of the high-level keychain intervals is sufficiently long to allow the use of a single keychain to cover the entire lifetime of the constrained receivers.

The next step is constructing the two low-level keychains that will be embedded inside each time interval of the high-level keychain. The two low-level keychains are in offset alignment with each other, and the generation of the consecutive low-level keychains is in accordance with the process of the enhanced Inf-TESLA protocol described in [15]. The high-level and the low-level keychains are linked to one another through pseudo-random functions, wherein low-level keys can be re-generated from high-level keys. To reduce the computational demands of the two different pseudo-random functions and generate last two keys of the low-level keychains, two salt values are used, each for a low-level keychain element to be added to $K_{i+1}\;$before the hashing process. The salt values are fixed and are sent in the synchronization message when the connection is established between the sender and the receiver. The low-level key elements are generated as follows:

(3) $$K_{i,n}=F_{01}\left(S_{1}(K_{i+1}\right))\;$$

(4) $$K_{i+1,n}=F_{01}\left(S_{2}(K_{i+1}\right))\;$$

where $K_{i,n}$ and $K_{i+1,n}$ are the last key elements of the two low-level keychains at time intervals i and i+1, respectively, $K_{i+1}$ is the high-level key at time interval i+1, $F_{01}$ is the pseudo-random hash function, and $S_{1}$ and $S_{2}$ are different salt values used to differentiate the generation of low-level key elements. Once the last key elements of the low-level keychains are generated, the hash function $F_{1}\;$is used to generate the rest of the low-level keychain elements. The next step is the construction of the transmitted packets by the sender. The Multilevel-µTESLA protocol generates CDM packets, which act as second synchronization schedule packets sent in the high-level keychain for the low-level keychain. CDM packets are used to distribute the important information including the commitment low-level keys of two-time intervals ahead, MAC value of the commitment key, and high-level key used to authenticate the previous CDM packet. The new Hybrid TLI-µTESLA protocol contains two low-level keychains, indicating that the original formation of the CDM packet in Equation (2) is modified to transmit two low-level commitment keys, for the alternating keychains, and their MAC value as follows:

(5) $$CDM_{i}=i\left|\left(K^{1}{}_{i+2,0},K^{2}{}_{i+2,0}\right)\right|MAC\;K_{i}\left(i|K^{1}{}_{i+2,0},K^{2}{}_{i+2,0}\right)K_{i-1}\;$$

where $CDM_{i}$ is the CDM packet sent during ith time interval, $K^{1}{}_{i+2,0},K^{2}{}_{i+2,0}$ are the low-level commitment keys for the first and the second alternating keychains in the time interval i+2, respectively, $K_{i}$ is the high-level key used to protect the packets sent at ith time interval, ${\mathrm{MAC}\;K}_{i}$ is the MAC value of the low-level commitment keys that are hashed using the high-level key $K_{i}$, $K_{i-1}$ is the high-level key used to authenticate the previous CDM packet sent during i−1 time interval, and | is the concatenation process. To avoid loss of important information sent by the CDM packets, the sender will randomly send several copies of $CDM_{i}$ packet during the ith time interval, and these copies are sent at random time instants and do not follow a regular timing pattern between the copies, as suggested by the Multilevel-µTESLA protocol.

The final step is to generate the broadcasting message packets to be sent in the two low-level keychains that follow the enhanced Inf-TESLA protocol. We chose the alternating mode of exchanging the keychains to send the message packets, where one keychain is responsible for the odd time intervals and the other keychain is responsible for the even time intervals. At the receiver side, check points are implemented before authenticating the keys and the message. First, the receiver should authenticate the first CDM packet that is received and save the important commitment keys related to the low-level keychains. Any additional CDM packets related to the same time interval will be dropped immediately to avoid buffer overflow. Second, the receiver should check if the received low-level packet is not within the disclosure delay time, otherwise it is considered a malicious packet and must be dropped. Further, the receiver will authenticate the previously buffered message packet by checking the currently received key with the last saved key or the commitment key of the corresponding low-level keychain. The MAC value of the buffered packet will then be computed using the currently received key and message, and the resulting value is compared with the previously buffered value. If the last element in the low-level chain is dropped, it can be regenerated using the high-level key based on Equations (3) and (4).

## 4. Handling Exceptional Broadcasted Packets in the Multilevel-µTESLA Protocol

An unaddressed problem in the Multilevel-µTESLA protocol is the authentication of the first and the last packets of the high-level and low-level keychains, where we proposed efficient solutions that do not increase the overall computational demands in the system and implemented them in the Hybrid TLI-µTESLA protocol. The exceptional cases are discussed as follows:

### 4.1. Handling Low-Level Exceptional Packets

#### 4.1.1. Handling the First d Packets

**Figure 3:**
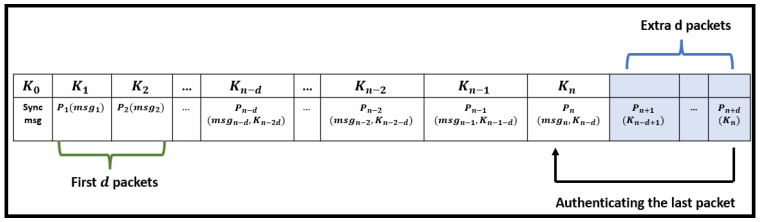
Handling the first and the last exceptional low-level packets in the Hybrid TLI-µTESLA Protocol.

As stated previously in the manuscript, one of the characteristics of the TESLA protocols is the asymmetric property, which is verified by hiding the key responsible for protecting the broadcasted packet for a certain pre-determined disclosure delay time d, until the key is revealed by the sender for authentication purpose. However, during the first d time intervals, no key element should be disclosed within the broadcasted packet because the packets are still within the disclosure time delay. Hence, the structure of the first low-level d packets (which is composed of message, MAC value and the disclosed key) is now composed of the message and MAC value, as shown in Figure 3.

#### 4.1.2. Handling the Last d Packets

As the broadcasting process of the low-level packets is completed, the last d packets received by the receiver remained buffered without being authenticated. The reason behind this problem is related to the structure of the last packets. For example, the last packet $P_{n}$ in Figure 3 is composed of message ${\mathrm{msg}}_{n}$, MAC value $MAC_{n}$, and the key $K_{n-d}$ that is used to authenticate the previously buffered packet $P_{n-d}$. This means that any packet received after $P_{n-d}$ will be buffered unnecessarily without being authenticated because the sender did not broadcast the corresponding keys. To overcome this, we proposed in [15] the addition of d extra packets to the low-level keychains, wherein these packets will only broadcast the necessary keys to authenticate the previously buffered packets, as shown in Figure 3. This solution is applied to all variant TESLA protocols, including Hybrid TLI- µTESLA protocol without affecting the overall computational overhead, mainly because the number of d packets added are much smaller compared to the size of the keychain.

### 4.2. Handling High-Level Exceptional Packets

#### 4.2.1. Handling the First Two CDM Packets

As stated previously, the CDM packet is responsible for broadcasting the commitment keys of the low-level keychains, two time intervals ahead. If we consider generating the first two CDM packets, it will be represented as follows:

(6) $$CDM_{0}=0\left|\left(K^{1}{}_{2,0},K^{2}{}_{2,0}\right)\right|MAC\;K_{0}(0|K^{1}{}_{2,0},K^{2}{}_{2,0})\;$$

(7) $$CDM_{1}=1\left|\left(K^{1}{}_{3,0},K^{2}{}_{3,0}\right)\right|MAC\;K_{1}(1|(K^{1}{}_{3,0},K^{2}{}_{3,0})|K_{0}\;$$

It is observed that $CDM_{0}$ does not have any high-level key to be disclosed yet, which should be considered as an exceptional broadcasted packet. Moreover, it is observed that in $CDM_{1}$, the commitment key of the high-level keychain $K_{0}$ is disclosed to authenticate the previous $CDM_{0}$ packet. This disclosure is considered very risky as it increases the chance of a brute-force attack, where the packet becomes vulnerable to being sniffed by an attacker, which can lead to breaking the high-level and the low-level keychains of the Hybrid TLI-µTESLA protocol. Therefore, $K_{0}$ should not be disclosed in any CDM packet as it is sent secretly in the first synchronization packet, during the initialization of the connection between the sender and the receiver. Thus, the receiver can successfully authenticate $CDM_{0}$ without the need to disclose $K_{0}$ in $CDM_{1}$. Hence, the modified $CDM_{1}$ packet is represented as follows:

(8) $$Modified\;CDM_{1}=1\left|\left(K^{1}{}_{3,0},K^{2}{}_{3,0}\right)\right|MAC\;K_{1}(1|(K^{1}{}_{3,0},K^{2}{}_{3,0})\;$$

**Figure 4:**
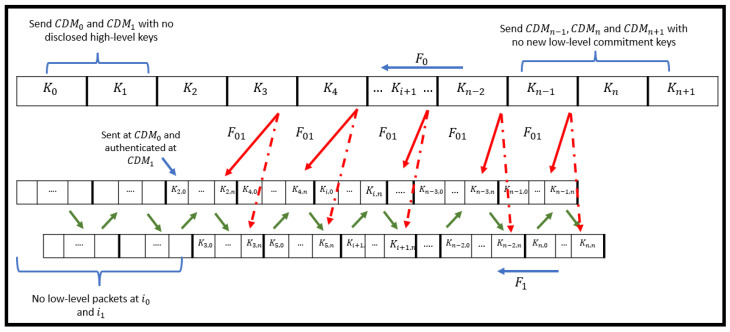
Final Structure of the Hybrid TLI-µTESLA Protocol including the exceptional packets in the high-level and the low-level keychains.

It should be noted that based on the structure of the first CDM packet that broadcasts the first commitment keys of the low-level keychains to be used at (i=2) time interval, the sender will only send CDM packets to the receiver in the first two high-level intervals (i=0, and i=1), and at i=2, the sender will start sending the message packets in addition to CDM packets, as shown in Figure 4.

#### 4.2.2. Handling the Last CDM Packets

The Multilevel-µTESLA protocol has considered a link between the high-level and low-level keychains based on the condition that the high-level keychain should be one interval ahead of the low-level keychains [14]. In other words, if the last element of the high-level keychain is $K_{n+1}$, the last interval in the low-level keychain should be $K_{n,n}$. The CDM packet is responsible for sending the commitment keys of the low-level keychains two time intervals ahead. Thus, based on the previous conditions in the Hybrid TLI-µTESLA protocol, the CDM packet responsible for sending the last commitment keys of the low-level keychains is as follows:

(9) $$CDM_{n-2}=n-2\left|\left(K^{1}{}_{n,0},K^{2}{}_{n,0}\right)\right|MACK_{n-2}\left(1|K^{1}{}_{n,0},K^{2}{}_{n,0},\right)|K_{n-3}$$

According to the previous equation, the CDM packets in the last three high-level intervals (i=n−1,i=n, and i=n+1) will be modified such that the sender only sends the CDM packets that contain the required high-level keys to authenticate the previous CDM packets. Figure 4 summarizes the overall construction of the Hybrid TLI-µTESLA protocol keychains and its exceptional packets.

## 5. Comparative Analysis of the Proposed Hybrid TLI-µTESLA Protocol and the Other Variant TESLA Protocols

We performed simulation analysis using Java software and compared the performance of the proposed Hybrid TLI-µTESLA protocol and other variant TESLA protocols, including the original TESLA, enhanced Inf-TESLA, and Multilevel-µTESLA protocols, given that they are closely related to the structure of the Hybrid TLI-µTESLA protocol. The performance evaluation is based on the study of the time complexity analyses of the protocols and computational overhead consumed at the sender and receiver sides. We also analyzed the cyber-security properties that are realized using the Hybrid TLI-µTESLA protocol. The previous analyses were performed using two different hash functions for the first time, SHA-2 and SHA-3, to study the impact of the hash function on the overall performance of variant TESLA protocols. The simulation setups include a common scenario between a receiver establishing a secured connection with the server and receiving encrypted messages using variant TESLA protocols. The disclosure time delay is assigned to be 2 interval durations and the UDP buffer size is 1024 bits. The number of simulations performed is 50 simulations, where the average is considered for a reliable comparison. The next section includes a detailed discussion on each performance evaluation.

### 5.1. Performance Analysis at the Sender

This section discusses the computational overhead at the sender side based on the evaluation of the time complexity of the algorithm and generation time of the keychains in the Hybrid TLI-µTESLA protocol and comparison with other TESLA protocol variants.

#### 5.1.1. Time Complexity Analysis

In this section, we analyzed the time complexity of the Hybrid TLI-µTESLA and other TESLA variant protocols from the sender’s perspective. We considered the Big O notation because it quantifies the amount of time required by the algorithm to be accomplished as a function of the input length [21,22]. The time complexity of the algorithm is related to the most time-consuming parts, such as a repetitive operation that is controlled by an input size which can be changed according to the application specifications. Therefore, we focused on the loop operation related to the generation of the keychains using SHA-2 hash function, and we changed the required size accordingly. We varied the keychain length between 100 and 1000 keys and recorded the generation time of the related keychains. The number of simulative iterations was 30, and we calculated the average per each keychain length based on this number. A curve fitting was conducted to best describe the resulting data.

**Figure 5:**
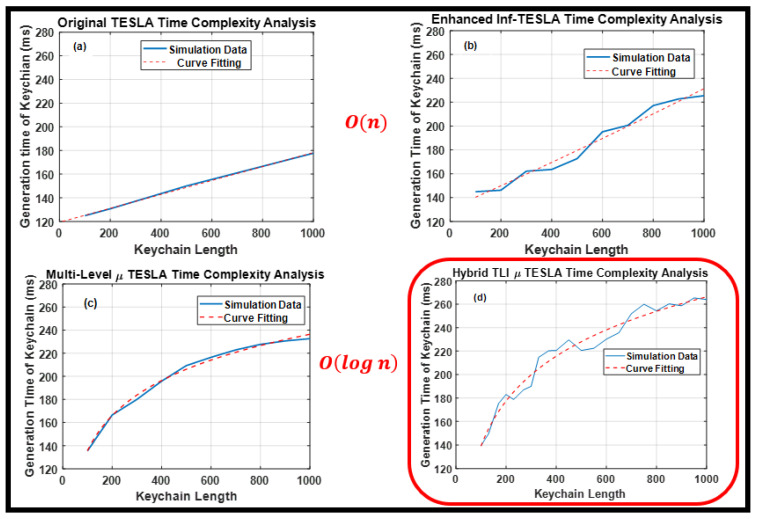
Time complexity analysis of (**a**) Original TESLA, (**b**) Enhanced Inf-TESLA, (**c**) Multilevel-µTESLA, and (**d**) Proposed Hybrid TLI-µTESLA protocols.

Figure 5 shows the simulation data and curve fitting results for (a) Original TESLA protocol, (b) enhanced Inf-TESLA protocol, (c) Multilevel-µTESLA protocol, and (d) Hybrid TLI-µTESLA protocol. It was observed that the generation time of the keychain for the original TESLA and the enhanced Inf-TESLA protocols increases linearly with the increase in the number of keys required in the chain. Consequently, the linear relationship in the Big O notation is described by O(n), where n is the number of keys in the keychain. The linear time complexity is considered an acceptable level of computational demand for constrained devices. The similarity in the results between the original TESLA and the enhanced Inf-TESLA is relatively reasonable because the enhanced Inf-TESLA protocol includes two keychains that are parallel to each other and operate using alternating mode, wherein one key is used from each chain per transmitted packet.

Regarding the Multilevel-µTESLA protocol and Hybrid TLI-µTESLA protocol, we observed that with the increase in the number of keys required in the chain, the generation time of the keychain increases slowly, following a logarithmic behavior O(log n). The logarithmic behavior is considerably powerful because the complexity of log(n) represents the computational demands that do not increase with the input size. The similarity between the Multilevel-µTESLA protocol and Hybrid TLI-µTESLA protocol is reasonable as the overall structure of high-level and low-level keychains is the dominant in both the algorithms. The inclusion of the two parallel low-level keychains in the Hybrid TLI-µTESLA protocol is the reason behind the zigzag behavior of the simulation results in Figure 5d. We can therefore observe that the Hybrid TLI-µTESLA showed the best time complexity performance among the TESLA protocols, following a logarithmic behavior that brings the computational demands to a minimum level.

#### 5.1.2. Generation Time of the Keychain vs. Changing the Key Length

**Figure 6:**
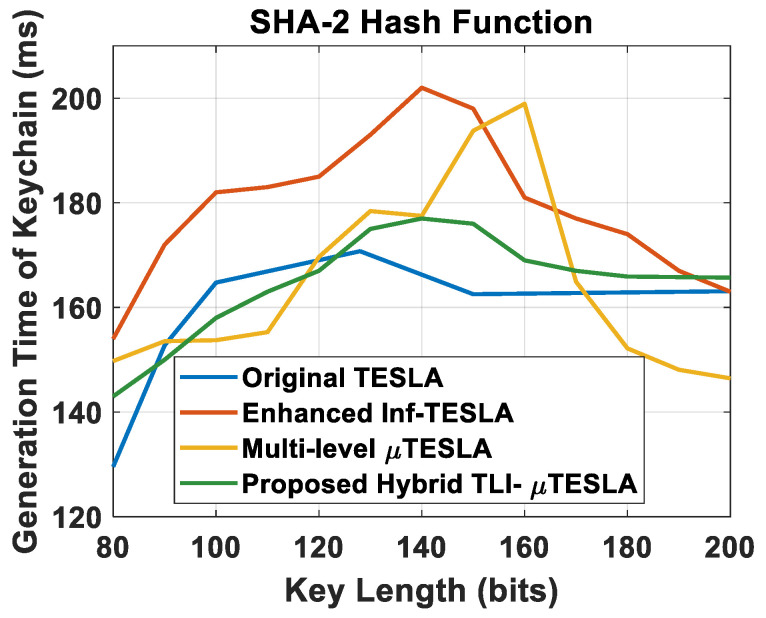
Simulation results of original TESLA protocol (blue line), enhanced Inf-TESLA protocol (red line), Multilevel-µTESLA protocol (yellow line), and Proposed Hybrid TLI-µTESLA protocol (green line), demonstrating the computational overhead at the sender side during the generation of a keychain with the change in the key size using SHA-2 hash function.

Our next analysis is based on studying the impact of changing the key length on the generation time of the keychain for a fixed keychain length. The key length was varied between 80 and 200 bits, which is an acceptable range of key length sizes to be applicable for constrained devices to still achieve a reasonable cybersecurity level. The number of simulative iterations were 50. The average for each key length was considered, and the hash function used was SHA-2. The results of the comparison between the Hybrid TLI-µTESLA and the variant TESLA protocols are shown in Figure 6.

From the results, we observe that the generation time of the keychain in Hybrid TLI-µTESLA at the sender side is closer to the original TESLA protocol. The turning point is however similar to the enhanced Inf-TESLA protocol at 140-bit key length size. We also observe that as the key length increases, the Hybrid TLI-µTESLA keychain generation time decreases until it converges to approximately 165 ms, which is close to that of the Inf-TESLA and Original TESLA protocols. This observation helps select a sufficiently large key size with the aim of increasing the robustness against Brute-force attacks and reducing the probability of breaking the keychain by attackers, without increasing the overall computation overhead of the network. Moreover, the overall range of values of the Hybrid TLI-µTESLA protocol is within the minimal values compared to other variants of TESLA protocol, indicating an enhancement over other protocols and a low computational cost on the sender side in terms of the low generation time of three different keychains.

### 5.2. Performance Analysis at the Receiver

We further considered the analysis of the computation overhead of the Hybrid TLI-µTESLA protocol at the receiver side, wherein we computed two critical parameters: the generation time of the MAC value and the authentication time of the packets. We have provided a detailed discussion about each computation in the following section.

#### 5.2.1. Generation Time of the MAC Value vs. Changing the Key Length

**Figure 7:**
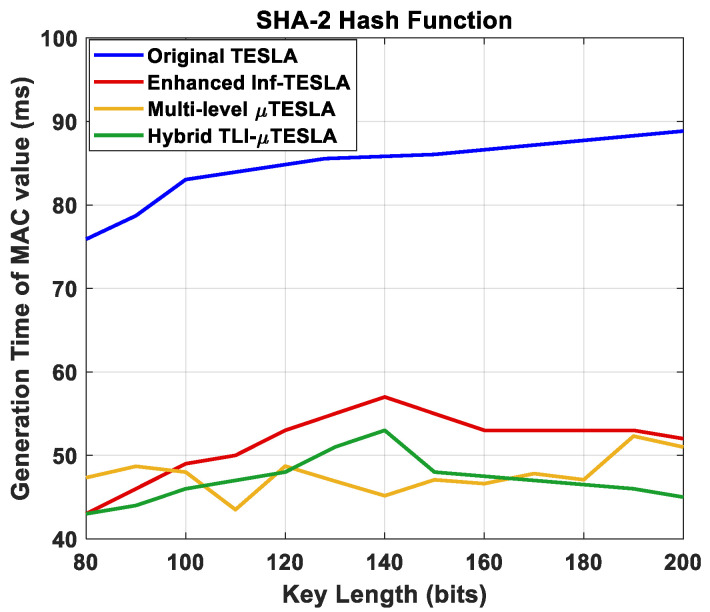
Simulation results of the original TESLA protocol (blue line), enhanced Inf-TESLA protocol (red line), Multilevel-µTESLA protocol (yellow line), and Proposed Hybrid TLI-µTESLA protocol (green line), demonstrating the computational overhead at the receiver side during the generation of the MAC value with the change in the key size to authenticate the received packet using SHA-2 hash function.

We studied the impact of changing the key length on the generation time of the MAC value, which is among the critical processes at the receiver side to authenticate the confidentiality and the integrity of the packets and their source. The key length was varied between 80 and 200 bits, and the number of simulative iterations was 50, where we considered the average value per key length. The simulations were performed using SHA-2 hash function. Figure 7 shows the generation time of the MAC value with a comparison between the Hybrid TLI-µTESLA and the variant TESLA protocols.

The results revealed that the generation time of the MAC value for the Hybrid TLI-µTESLA protocol is lower than that of the Original TESLA and the enhanced Inf-TESLA protocols, and it is comparable to the Multilevel-µTESLA protocol. Moreover, we can observe the impact of Multilevel-µTESLA protocol in reducing the authentication time of the packets and providing nearly immediate authentication process inherited to the Hybrid TLI-µTESLA protocol. We also observed that the overall change in the generation time of the MAC values is within a small range (~5 ms) for the Hybrid TLI-µTESLA protocol, indicating the stability of performance in the algorithm. Another important finding is that the generation time of the MAC value is unaffected by the change in the key length in Hybrid TLI-µTESLA protocol. This further supports the applicability of choosing large key size to increase the robustness of the network against Brute-force attacks, without increasing the computational overhead of the system.

#### 5.2.2. Authentication Time of the Packets vs. Changing the Key Length

**Figure 8:**
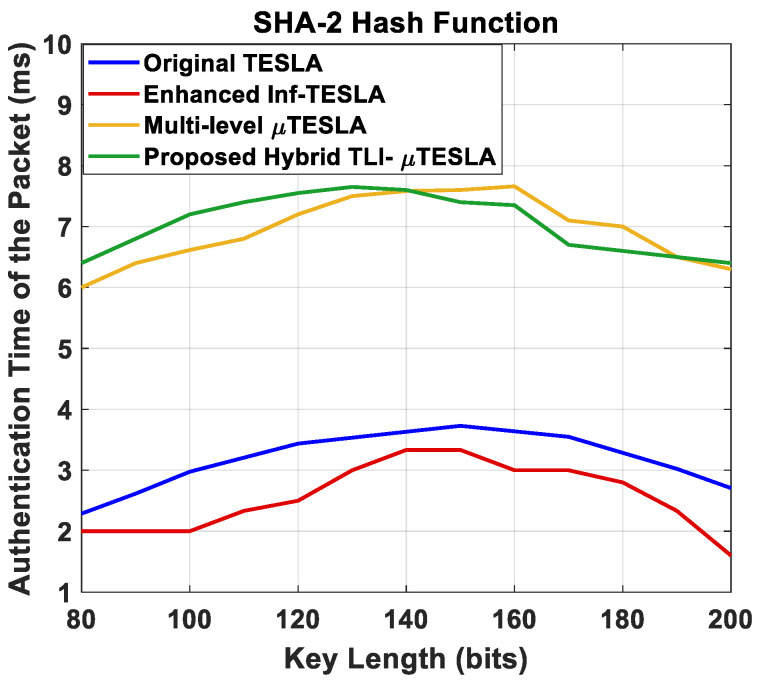
Simulation results of the original TESLA protocol (blue line), enhanced Inf-TESLA protocol (red line), Multilevel-µTESLA protocol (yellow line), and Proposed Hybrid TLI-µTESLA protocol (green line), demonstrating the computational overhead at the receiver side during the authentication time of the received packets with the change in the key size using SHA-2 hash function.

We analyzed the authentication time of the packets with respect to the change in the key length, which is another critical point of evaluating the integrity of the data at the receiver side. The key length was varied between 80 and 200 bits, and the number of simulative iterations was 50, where we took the average per key length. The simulations were performed using SHA-2 hash function. Figure 8 shows the authentication time of the packets, based on a comparison between the Hybrid TLI-µTESLA and the variant TESLA protocols.

The first observation is that the authentication time behavior in Hybrid TLI-µTESLA protocol is similar to that in the variant TESLA protocols, which indicates a form of systematic behavior between the variant protocols. We also observed that the range of authentication time values in the Hybrid TLI-µTESLA protocol is similar to the Multilevel-µTESLA protocol because both should authenticate two types of packets: the low-level message packets and the high-level CDM packets. Hence, we extracted two types of packets in the Hybrid TLI-µTESLA and the Multilevel-µTESLA protocols to further investigate the authentication time behavior, and we compared them with the other variant TESLA protocols based on the previous comparison of the authentication time of the low-level message packets.

**Figure 9:**
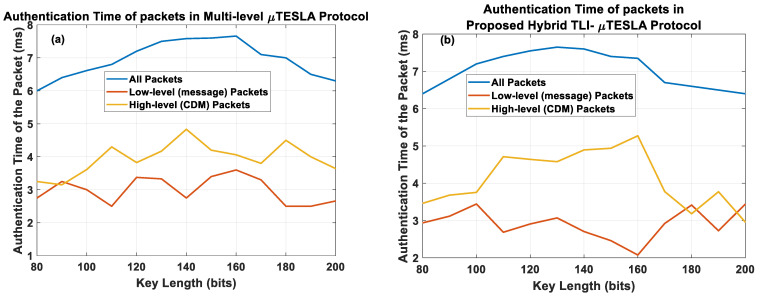
Simulation results showing the composition of the authentication time of the low-level (message) packets (red line), authentication time of the high-level CDM packets (yellow line), and overall authentication time of the packets (blue line) in (**a**) Multilevel-µTESLA and (**b**) Proposed Hybrid TLI-µTESLA protocols using SHA-2 hash function.

**Figure 10:**
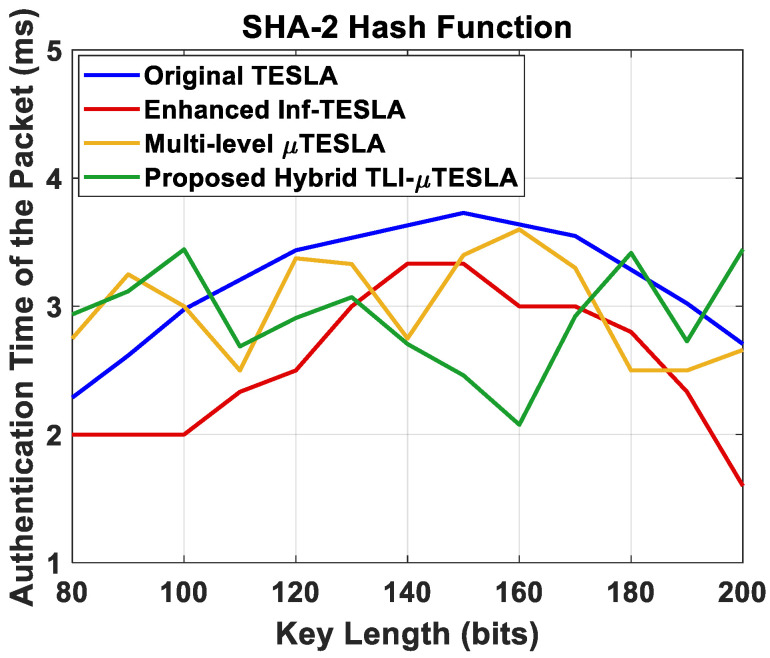
Simulation results of the original TESLA protocol (blue line), enhanced Inf-TESLA protocol (red line), Multilevel-µTESLA protocol (yellow line), and Proposed Hybrid TLI-µTESLA protocol (green line), demonstrating the authentication time of the low-level (message) packets with the change in the key size using SHA-2 hash function.

Figure 9a shows the composition of the authentication time of the packets in the Multilevel-µTESLA protocol, and Figure 9b shows the composition of the authentication time of the packets in the Hybrid TLI-µTESLA protocol. It is observed that the overall behavior of the Hybrid TLI-µTESLA protocol is similar to that of the Multilevel-µTESLA protocol as they both share the same structure and possess a high-level and low-level keychain. Furthermore, the authentication time of the low-level packets in both protocols is less than that of the high-level packets, which is mainly because of the short time duration of the low-level keychains and their purpose of speeding the authentication process of the messages between the sender and the receiver. However, we noticed that the authentication time of the high-level CDM packets is relatively higher in the Hybrid TLI-µTESLA protocol compared to the Multilevel-µTESLA protocol because of the need to authenticate two commitment keys related to the low-level parallel keychains in the Hybrid TLI-µTESLA protocol. After extracting the high-level and the low-level packets from the Hybrid TLI-µTESLA and the Multilevel-µTESLA protocols, we used the low-level message packets from both protocols for comparison with the original TESLA and the enhanced Inf-TESLA protocols. Figure 10 shows the overall authentication time of the low-level message packets for all variant TESLA protocols.

The results revealed that the authentication time of the low-level packets in the Hybrid TLI-µTESLA and Multilevel-µTESLA protocols are unaffected by the change in the key length, indicating good network stability. Moreover, the variation of the authentication time of the low-level packets in the Hybrid TLI-µTESLA protocol is within (0.5 ms–1 ms), which is less than that of the other TESLA protocols (which varies around 1.5 ms) and similar to that of the Multilevel-µTESLA protocol, indicating the significant impact of the Multilevel-µTESLA protocol on Hybrid TLI-µTESLA based on the reduction in the authentication time of the packets and nearly immediate authentication process. These results ensure that by using a relatively large key size, the authentication time of the packets would be sufficiently low for a satisfactory lifetime of the network.

### 5.3. Performance Analysis Using SHA-3 Hash Function

In this section, we studied the impact of the change in the hash function from SHA-2 to SHA-3 on the overall performance of the TESLA protocols. We intended to analyze the ability of our proposed Hybrid TLI-µTESLA protocol to handle quantum attacks and cater to forthcoming 5G and 6G IoT network generations. We studied the impact of SHA-3 hash function on the computational overhead at the sender side by evaluating the generation time of the keychain with respect to the change in the key length; while we monitored the computational overhead at the receiver side by evaluating the generation time of the MAC value and authentication time of the packets with respect to the change in the key length.

**Figure 11:**
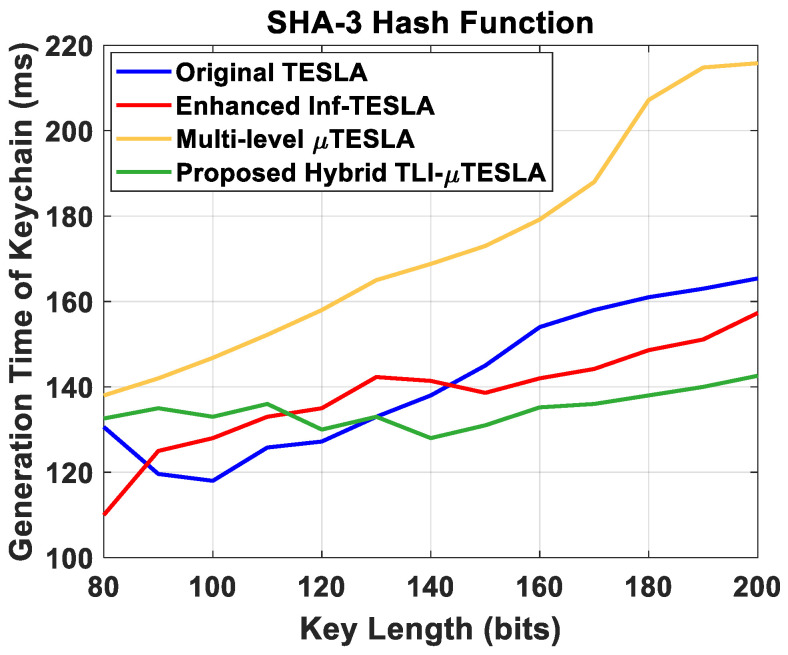
Simulation results of the original TESLA protocol (blue line), enhanced Inf-TESLA protocol (red line), Multilevel-µTESLA protocol (yellow line), and Proposed Hybrid TLI-µTESLA protocol (green line), demonstrating the computational overhead at the sender side during the generation of a keychain with the change in the key size using SHA-3 hash function.

Figure 11 shows the generation time of the keychain at the sender side with respect to change in the key length. The results reveal that the implementation of SHA-3 hash function has a positive impact on the original TESLA, the enhanced Inf-TESLA, and the Hybrid TESLA protocols. By comparing Figs. 11 and 6, we notice that the overall generation time of the keychains in original TESLA and enhanced Inf-TESLA protocols decreased when using SHA-3 hash function, but their stabilization values are similar when the SHA-2 hash function is used. However, the generation time of the keychains in Multilevel-µTESLA increased when using SHA-3 hash function because of the increase in the computational demands of the hash function and complex structure of the protocol. Regarding the Hybrid TLI-µTESLA protocol, we observe that the overall generation time of the keychain reduced when the SHA-3 hash function was used, and it remained almost unaffected by the change in the key-length value, which indicates the significant impact of the enhanced Inf-TESLA protocol on the Hybrid TLI-µTESLA protocol in reducing the computational demands of the network.

**Figure 12:**
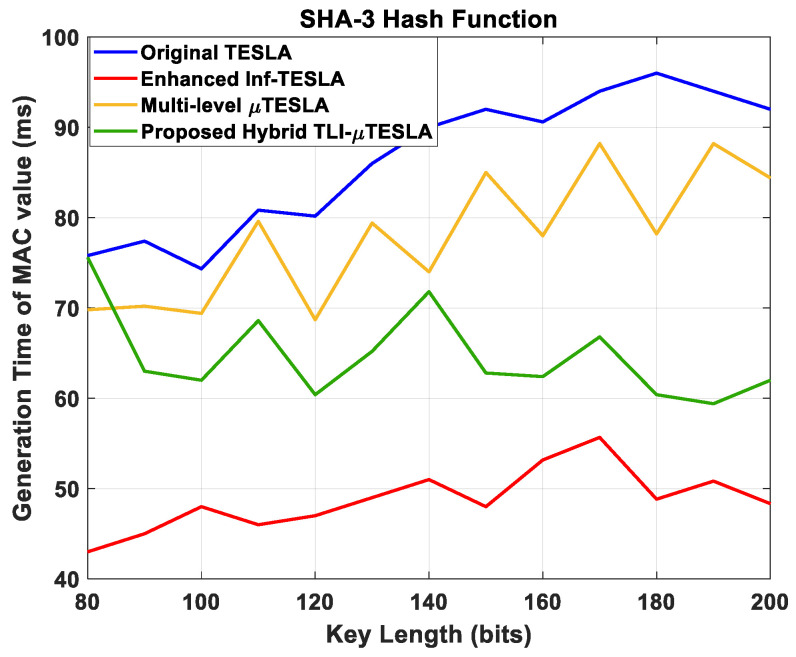
Simulation results of the original TESLA protocol (blue line), enhanced Inf-TESLA protocol (red line), Multilevel-µTESLA protocol (yellow line), and Proposed Hybrid TLI-µTESLA protocol (green line), demonstrating the computational overhead at the receiver side during the generation of the MAC value with the change in the key size to authenticate the received packet using SHA-3 Hash Function.

Figure 12 shows the generation time of the MAC value at the receiver side with respect to the change in the key length. The results reveal that the implementation of SHA-3 hash function has a significant impact on the Multilevel-µTESLA and Hybrid TLI-µTESLA protocols, resulting in an increase in the generation time of the MAC value compared to when the SHA-2 hash function was used, in Figure 7. This is because the SHA-3 hash function software operations are slower than those of SHA-2 hash function as it is associated with two stages; absorption of the input data into four algorithms with different hash functions and squeezing process into two extendable output functions to perform domain hashing, randomized hashing, and stream encryption and to generate MAC addresses of the final output [23].

Moreover, the Multilevel-µTESLA and Hybrid TLI-µTESLA protocols are associated with the high-level keychain that transmit CDM packets, which require considerable time to authenticate the MAC value and the commitment keys of the low-level keychains. The overall increase in the generation time of the MAC value in the Multilevel-µTESLA and Hybrid TLI-µTESLA protocols when using SHA-3 hash function is approximately 20–25 ms, which is considered as an acceptable level of computational demands in constrained devices. It can also be implied that the impact of SHA-3 hash function on the original TESLA and the enhanced Inf-TESLA protocols is minimal, and thus, the performance is nearly similar to the case when SHA-2 hash function was used. This is because of the absence of high-level keychain structure that is available in the Multilevel-µTESLA and Hybrid TLI-µTESLA protocols, wherein significant time is consumed to authenticate the MAC value and commitment keys of the low-level keychains.

**Figure 13:**
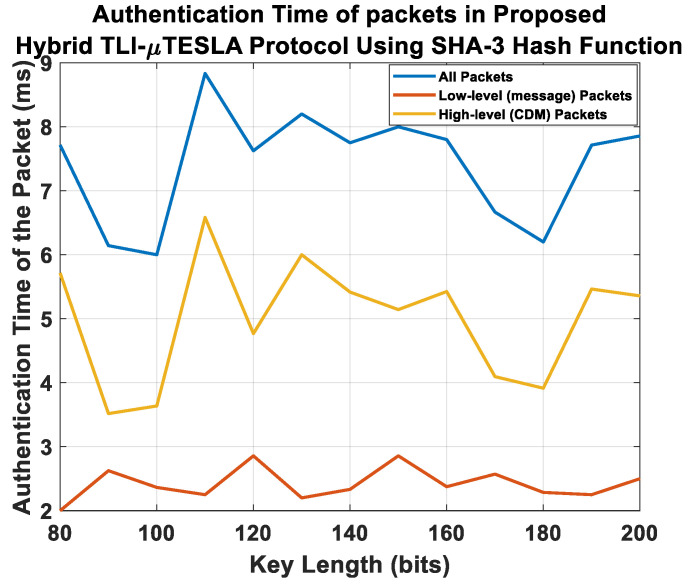
Simulation results showing the composition of the authentication time of the low-level (message) packets (red line), authentication time of the high-level CDM packets (yellow line), and overall authentication time of the packets (blue line) in the Proposed Hybrid TLI-µTESLA protocols using SHA-3 Hash Function.

**Figure 14:**
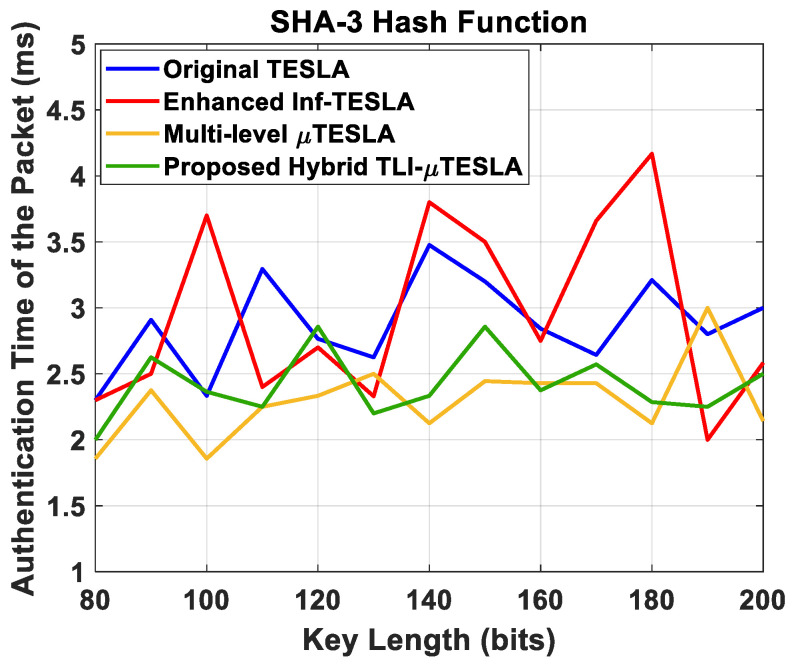
Simulation results of the original TESLA protocol (blue line), enhanced Inf-TESLA protocol (red line), Multilevel-µTESLA protocol (yellow line), and Proposed Hybrid TLI-µTESLA protocol (green line), demonstrating the authentication time of the low-level (message) packets with the change in the key size using SHA-3 Hash Function.

Figure 13 shows the composition of the authentication time of the packets in the Hybrid TLI-µTESLA protocol using SHA-3 hash function. Figure 14 shows the authentication time of the low-level packets for all TESLA variant protocols using SHA-3 hash function. It was observed that a significant portion of the authentication time of the packets in the Hybrid TLI-µTESLA protocol occurs during the authentication of the CDM packets, due to the packet structure and content of the information that should be authenticated. We used the authentication time of the low-level packets in the Hybrid TLI-µTESLA protocol for comparison with the authentication time of the low-level packets of the other TESLA protocols.

The results in Figure 14 reveal that the effect of using SHA-3 hash function has a relatively small impact on the authentication time of the packets, and it increases to approximately 0.5–1 ms for all variant TESLA protocols, when compared to Figure 10. However, the authentication time of the low-level packets is unaffected by the change in the key-length values for all TESLA protocols, indicating the stability of the network regardless of the type of hash function used. Moreover, the authentication time of the low-level packets in the Hybrid TLI-µTESLA and Multilevel-µTESLA protocols are minimal compared to the original TESLA and the enhanced Inf-TESLA protocols, indicating the impact of the Multilevel-µTESLA protocol on the Hybrid TLI-µTESLA protocol in reducing the authenticating time of the packets and enabling nearly immediate authentication.

### 5.4. Cybersecurity Analysis of the Hybrid TLI-µTESLA Protocol

In this section, we analyze the cybersecurity attacks and study the Hybrid TLI-µTESLA protocol robustness and ability to mitigate authentication risks including Masquerading, Man-in-the-Middle, Modification, and DoS through replay traffic. We also considered theoretical comparison between the proposed Hybrid TLI-µTESLA protocol and other variant TESLA protocols by updating the table mentioned in [15] that discusses the advantages and disadvantages of TESLA protocols on IoT constrained devices.

The first attempted attacks that are faced under any protocol are the Masquerading and Man-in-the-Middle attacks, where an attacker can listen to the broadcasted packets and tries to forge its key while assuming to be an authorized user in the network. This attempt will allow the attacker to have a complete control of the next broadcasting packets and enforce the network to behave maliciously. To overcome these risks, the Hybrid TLI-µTESLA protocol has two parallel low-level keychains with very short time intervals that work in an alternating mode, wherein a key from each chain is used alternatively among the transmitted packets. Therefore, the ability of an attacker to break two keychains in a significantly short time duration is nearly impossible. Moreover, a high-level keychain is associated with the Hybrid TLI-µTESLA protocol, which periodically sends new commitment keys of the low-level keychains for their next time intervals. Thus, the ability of an attacker to break three different keychains in a short time interval is nearly impossible.

The next possible attempted attack is the modification attack, wherein an attacker listens to the broadcasted packets sent at the end of the keychain and modifies the keys, message, and MAC values to break the chains of the next time interval. However, substituting the packet during the disclosure delay (d) of the protocol (where d is a secret parameter sent only between the sender and receiver during their synchronization packet) is a violation of the protocol, which will result in the packet being dropped immediately. This is because the receiver first checks the forged key against the low-level commitment keys of the new keychains that are secretly sent in the Hybrid TLI-µTESLA protocol using high-level CDM packets two time intervals prior. If the forged key is not authenticated by any of the commitment keys, the packets are dropped. A final attempt by an attacker is to flood the network buffer with DoS and DDoS attacks and subsequently turn down the system. Such attacks are solved using the Hybrid TLI-µTESLA protocol by including a time stamp for each low-level packet. Once the disclosed key of the low-level packet is authenticated, any replays of the same packet containing the same key will be rejected to avoid replay attacks. This process is also implemented for high-level packets, such that if the same replica of the CDM packet that is previously authenticated is received, it will be immediately dropped to avoid buffer overflow. Table 1 summarizes the advantages and disadvantages of the Hybrid TLI-µTESLA protocol and other variant TESLA protocols.

## 6. Conclusions

In this study, we proposed a continuous, low-cost, and a highly secured protocol, named Hybrid TLI-µTESLA protocol, that supports immediate authentication. The proposed model combines and maximizes the functionality of our previously proposed enhanced Inf-TESLA protocol that provides continuous authentication and minimal overhead requirements between the sender and receiver, without reestablishing a synchronization packet with the Multilevel-µTESLA protocol. This protocol achieves immediate authentication of the transmitted packets between the sender and receiver with minimal computational demands while enhancing the cybersecurity level of the protocol. The Hybrid TLI-µTESLA protocol structure consists of a high-level keychain with CDM packets that are sent to transmit the commitment keys of the two low-level keychains that work in alternating mode, such that a key from each chain protects the packets in an alternative manner. The suggested structure improved the cybersecurity level of the protocol, making it highly robust against brute-force attacks because the probability of breaking three different keychains by an attacker during short time intervals is extremely low. We also revealed non-addressed problems in authenticating the first and the last d packets in the low-level keychains as well as last high-level CDM packets of the Multilevel-µTESLA protocol. We then proposed a considerable solution by modifying the structure of the exceptional packets and implemented it in the Hybrid TLI-µTESLA protocol to optimize the performance without increasing the computational demand of the protocol.

We conducted simulation analysis using JAVA to study the performance of the Hybrid TLI-µTESLA protocol and provided a comparison with the other TESLA protocols including the original TESLA, enhanced Inf TESLA, and Multilevel-µTESLA protocols. The comparative analysis included the study of the time complexity of the protocols and the generation time of the keychains at the sender’s side, while studying the critical authentication process such as MAC value and authentication of the packets at the receiver’s side with respect to the change in the key length. The results revealed that the Hybrid TLI-µTESLA protocol successfully combined the benefits of the previous TESLA protocols. For instance, the Hybrid TLI-µTESLA protocol enhanced the time complexity analysis (O log(n)) in a manner similar to the Multilevel-µTESLA protocol, which increases the lifetime of the network compared to the original TESLA and enhanced Inf-TESLA, whose time complexity is O(n). Moreover, the Hybrid TLI-µTESLA protocol improved the generation time of the keychain with minimal values similar to those of original TESLA protocol than of other variant TESLA protocols.

The generation time of the MAC value and the authentication time of the low-level packets using the Hybrid TLI-µTESLA protocol remained unaffected with the change in the key length at the receiver side, indicating good stability in the network regardless of the key size. Consequently, the Hybrid TLI-µTESLA protocol reduced the authentication time of the low-level packets and the generation time of MAC values. This was expected from the proposed model that consists of short time intervals, and it is affected by the Multilevel-µTESLA protocol, which enabled a nearly immediate authentication process with minimal computational demands. By implementing the enhanced Inf-TESLA protocol in the low-level chain of the Hybrid TLI-µTESLA protocol, we achieved a continuous authentication and synchronization between the sender and the receiver, which results in an increase in the cybersecurity level of the protocol without the need to reestablish a synchronization that drains the energy and the lifetime of the network. Furthermore, the Hybrid TLI-µTESLA protocol allowed larger key lengths to increase the resistance of quantum attacks without increasing the computation overhead on the receiver side.

We also performed a comparative analysis between the Hybrid TLI-µTESLA protocol and other TESLA protocols in terms of changing the hash function from SHA-2 to SHA-3 for the first time, to study the applicability of the Hybrid TLI-µTESLA protocol at higher levels of cybersecurity and for systems with newer IoT generations and higher data rates. The simulation results revealed that the implementation of SHA-3 hash function helped in improving the Hybrid TESLA protocol in terms of reducing the generation time of the keychains at the sender side, reducing the authentication time of the packets at the receiver side, and increasing the security level of the keychains compared to other TESLA protocols. These improvements were recorded and observed with respect to a minimal increase in the generation time of the MAC value because of the different structure and functions used in the SHA-3 hash function compared to the SHA-2 hash function.

Finally, we conducted a theoretical comparison between the Hybrid TESLA protocol and other TESLA protocols to study the robustness of the protocol against possible cyber-security attacks including Masquerading, Man-in-the-Middle, Modification, and Replay attacks. It was shown that the new structure of the Hybrid TESLA protocol that includes three different keychains and short time intervals in the low-level keychains helps in reducing the Brute-force, Masquerading, and Man-in-the-Middle attacks. Moreover, the checking criteria in the Hybrid TESLA protocol that do not allow alteration or addition of any packets within the disclosure delay time improves the resistance against Modification and Replay attacks. In addition, the ability of the Hybrid TESLA protocol to use large key lengths and implement SHA-3 hash function without increasing the computational demands on the networks offers it a superiority over other TESLA protocols, in terms of enhancing the lifetime and performance of the network and applicability with 5G and 6G IoT generations. In the future work, we intend to perform packet loss analysis on the Hybrid TESLA protocol and compare it with other TESLA protocols to study the performance of the protocols in a weak communication environment.

## Figures and Tables

**Table 1:** Comparison of Various TESLA Protocols.

Protocol	Advantages	Disadvantages	Time Complexity
TESLA [11]	Lightweight cryptography Simple implementation	DOS attacks Packet loss No scalability Noncontinuity in synchronization	O(n)
µTESLA [16]	Saves computation power and memory requirements Reduces the size of transmitted packets	No scalability Noncontinuity in synchronization	O(n)
Multilevel µTESLA [14]	Supports scalability Fault tolerance towards DOS attacks Provides immediate authentication	Increases buffering Increases computation overhead Noncontinuity in synchronization	Olog(n)
Infinite TESLA [11]	Reduces man-in-middle attack	No scalability Noncontinuity in synchronization	O(n)
Enhanced Infinite TESLA [12]	Reduces man-in-middle attack Reduces DOS attacks Continuous synchronization Saves computation power	Moderate computation overhead No immediate authentication	O(n)
Proposed Hybrid TLI-µTESLA	Reduces the authentication process of the packets Reduces man-in-middle attacks Reduces DOS attacks Continuous synchronization Saves computation power Supports Scalability	Moderate computation overhead	Olog(n)

## Abbreviations

**Abbreviation/Symbol**	**Explanation**
CDM	Commitment Distribution Message
d	Disclosure time delay
DoS	Denial of Service attack
$F_{0},\;F_{1},\;and\;F_{01}$	Pseudo-random functions
$K_{i,n}$	The last nth element of the low-level keychain in the ith time interval of Multilevel-µTESLA
$K_{i+1}$	the high-level key in the (i+1) time interval
$K^{1}{}_{i+2,0},K^{2}{}_{i+2,0}$	The commitment keys of the first and the second low-level keychains in the Hybrid TLI-µTESLA protocol
L	Length of High-level keychian
$MAC_{K_{i}}$	MAC value generated using $K_{i}$
n	Length of low-level keychains in the Multi-level µTESLA protocol
$S_{1}$ and $S_{2}$	Different Salt Values
